# Comparative Assessment of Agreement in Uniformity Analyses across Quality Control Software Platforms

**DOI:** 10.1055/s-0044-1795102

**Published:** 2024-11-19

**Authors:** Thasmeera T. Supramaniam, Muhammad Y. Udin, Marianie Musarudin

**Affiliations:** 1 School of Health Sciences, Health Campus, Universiti Sains Malaysia, Kelantan, Malaysia; 2 Department of Nuclear Medicine, Radiotherapy and Oncology, Hospital Pakar Universiti Sains Malaysia, Kelantan, Malaysia

**Keywords:** uniformity, ImageJ, NMQC Toolkit, NM Toolkit, SPECT

## Abstract

**Objective** In nuclear medicine, quality control (QC) activities adhere to international standards, yet their complexity can pose challenges. Gamma camera manufacturers have introduced integrated QC software, offering instantaneous results. However, the agreement of these automated processes with established protocols remains uncertain. This study aims to clarify this uncertainty by comparatively analyzing uniformity from various software solutions for a dual-head gamma camera.

**Methods** The study utilized integrated QC analysis software and three free QC analysis tools (IAEA-NMQC Toolkit, NM Toolkit, and Fiji) for uniformity analyses. Following the National Electrical Manufacturers Association standards, NEMA Standards Publication NU 1-2018, the intrinsic uniformity test was performed on a GE Discovery NM/CT 670 Pro system. Ten uniformity QC images underwent analysis with both integrated QC software and alternative software. Data agreements were tested using the Blant–Altman regression-based analysis.

**Results** Significant differences were observed in integral and differential uniformities ( *p* < 0.001). The central field of view (useful field of view) integral uniformity mean differences for NMQC Toolkit, NM Toolkit, and Fiji were 2.46% (2.34%), 2.44% (2.31%), and 2.56% (2.64%), respectively. Conversely, x-differential and y-differential uniformity mean differences were consistently under 2%. Regression-based analysis confirmed good agreement between computed values.

**Conclusion** The integrated QC software of Discovery NM/CT 670 Pro provides reliable uniformity analysis, aligned with the NEMA standards. Variations in computed values may stem from differences in pixel values and applied data corrections.

## Introduction

Nuclear medicine imaging retrieves details regarding the physiological function and radiopharmaceutical distribution within a specific organ. The reliability of NaI (Tl) based single photon emission computed tomography (SPECT) detectors is influenced by several factors, including potential errors arising from the conversion of gamma photons into electrical signals, leading to issues like photon loss, motion artifacts due to extended acquisition time, and increased radiation exposure. 1 Common sources of artifacts in SPECT imaging encompass nonuniformity in gamma camera detectors, center of rotation errors, misalignment of cameras in multidetector scanner systems, reconstruction error, patient movement, unintended uptake of radiotracers in other organs, and attenuation. 2 Among these factors, uniformity emerges as the most crucial gauge of SPECT performance, prompting daily performance evaluations. Even a minor flaw in detector uniformity, as minimal as a 3% disparity, can result in a noticeable artifact in the reconstructed image. 3 Notably, most issues linked to the integrity of the detector head, computer system, and hard copy device can be identified through the uniformity image. Gamma camera system nonuniformity typically manifests as conspicuous concentric ring artifacts, 4 5 often necessitating recalibration of the detectors. 5

In SPECT imaging, numerous physical parameters influence image uniformity, necessitating the consideration of each parameter before commencing imaging. Nonuniformities may stem from diverse factors, encompassing fluctuations in the pulse-height spectrum of the photo multiplier tubes (PMTs), 6 spatial nonlinearities, 7 and other issues such as incorrect energy settings, flawed linearity maps, poor PMT balance, and scintillation crystal hydration. 8 Consequently, achieving standardization and automation in SPECT proves challenging. Determining the threshold for gamma camera nonuniformity poses difficulty due to unknown reproducibility in quality control (QC) measurements and service adjustments. In nuclear medicine, sustaining optimal gamma camera performance typically demands stringent adherence to QC tests, often aligning with international standards sets by entities like the National Electrical Manufacturers Association (NEMA), American Association of Physicists in Medicine (AAPM), Society of Nuclear Medicine and Molecular Imaging (SNMMI), and European Association of Nuclear Medicine (EANM) protocols, widely recognized as reference guidelines. 9 Yet, implementing these protocols routinely face challenges due to their intricacies.

To alleviate the burdens associated with QC testing, manufacturers have introduced gamma camera systems equipped with integrated software for analyzing QC outcomes, facilitating immediate assessment of the instrument's performance parameters. Most vendor-specified or integrated QC analysis software systems are developed based on standardized QC protocols but optimized to suit the technological specifics of the systems, ensuring optimal functionality and results. For instance, certain gamma camera manufacturers integrate uniformity correction, alongside energy and linearity corrections, to rectify residual nonuniformity and collimator imperfections. 8 Furthermore, result analysis is often automated, presenting finalized outcomes without extensive elaboration, albeit lacking user configurability. 10 The complex computation applied to the data prior to perception can introduce additional uncertainties in various ways. 11 12 Previous studies have reported deviations in the agreement of built-in QC analysis software with standard QC protocols. 13 14 15 16 However, the rigorous assessment of the software's agreement remains lacking. Considering these aspects, it becomes imperative to evaluate the alignment between vendor-developed software and NEMA standards using independent analysis QC software.

Hence, to authenticate and ensure the reliability of the integrated QC analysis software developed by the manufacturer, this study aims to compare the intrinsic uniformity QC outcomes generated by our institution's integrated QC analysis software with the data derived from three distinct QC analysis software—both free and open/closed source—to ascertain their level of concordance. This study employed three separate QC software tools, previously cited in the literature, 13 17 18 in addition to the integrated QC software. These software packages encompass the NMQC Toolkit, NM Toolkit, and Image J (Fiji). Among these, Image J has been widely adopted in nuclear medicine image analysis and applications, likely owing to its open source nature. 19 20 21 22 23 24

## Materials and Methods

The entire data acquisition and analysis were carried out using an integrated SPECT/CT system (Discovery NM/CT 670 Pro, GE Medical System, United States) and its common user interface for the uniformity analysis. Three distinct, open/closed source of QC analysis software packages were employed to analyze the uniformity: IAEA-NMQC Toolkit version 1.0.14, 17 Fiji – ImageJ, 25 and NM Toolkit version 1.5.24. 26

### Intrinsic Uniformity Test

**Fig. 1:**
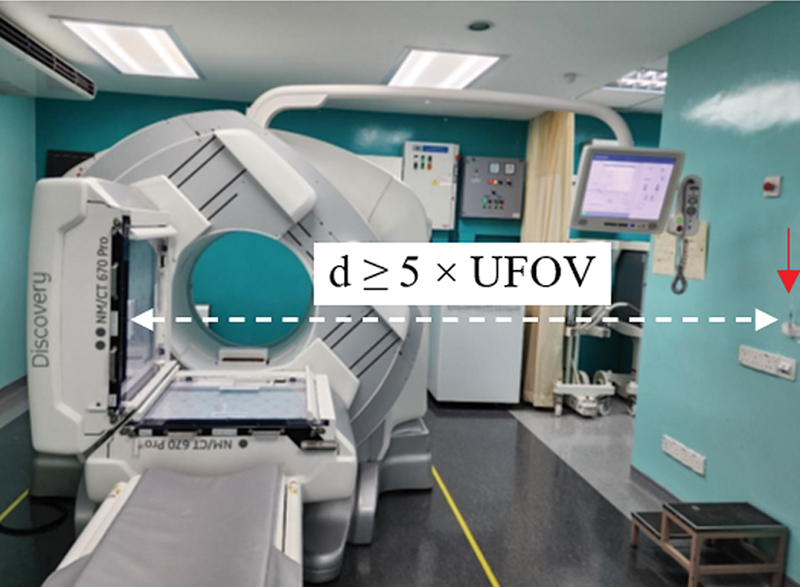
The intrinsic uniformity setup. ^99m^ Tc point source was positioned in a holder (pointed by the *red arrow* ) at a distance of ≥5 times the largest dimension of the uniform field of view (UFOV) of the detector, adhering to the NEMA guidelines.

In this investigation, we strictly adhered to the guidelines outlined in the NEMA Standards Publication NU 1-2018 when evaluating the intrinsic uniformity of the gamma camera system. 27 The collimators initially affixed to the gamma camera's detector heads were exchanged with decoy collimators. To prepare a ^99m^ Tc point source, we utilized a tuberculin syringe containing 800 μCi of ^99m^ Tc, 13 and positioned it at a location with a distance equivalent to five times the largest dimension of the useful field of view (UFOV) of the detector. 27 This positioning was precisely aligned with the central axis of the detector ( Fig. 1 ). During data acquisition, the process was programmed to halt at 20,000 kilocounts, employing an image matrix size of 128 × 128. The choice of the matrix size aligned with the NEMA guideline, stipulating that the flood field image should be stored in a matrix size generating pixel dimensions of 6.4 mm ± 30%. The energy window setting recommended by the manufacturer for ^99m^ Tc was employed, configured at 20%, symmetrically around the photopeak. In this study, the imaging acquisition was repeated for both detectors. After obtaining the uniformity images, the analysis was conducted using the integrated QC software within the SPECT system.

### Uniformity Analysis Using Free Open/Closed Source Software

Three distinct, free and open/closed source software applications—NMQC Toolkit, Fiji (ImageJ), and NM Toolkit—were employed. The first two software tools are available for download from their respective website: https://humanhealth.iaea.org and https://imagej.net/software/fiji/ . Meanwhile, the NM Toolkit is a closed source software provided for free, necessitating a request from its developer. 26 It is worth noting that the analyses performed by the NMQC Toolkit and NM Toolkit software align with the recommendations outlined in the NEMA Standards Publication NU 1-2018. Table 1 provides an overview of the software utilized in this research.

**Table 1:** The free, independent, open/closed QC analysis software tools used in this study

	Software		
	NMQC Toolkit	Fiji (Image J)	NM Toolkit
Accessibility	Free	Free	Free
Software type	Closed source	Open source	Closed source
Operating system compatibility/requirements	Windows, Mac OS X, Intel 32-bit or 64-bit with bundled Java	Windows, Mac OS X, and Linux in both 32-bit and 64-bit modes	Windows
DICOM image format support	Yes	Yes	Yes
Compliance with NEMA protocols	Yes	–	Yes
Available tests/tools for quality control	Yes	No a	Yes

Abbreviations: DICOM, Digital Imaging and Communications in Medicine; NEMA, National Electrical Manufacturers Association; QC, quality control.

a It is not specifically designed for quality control in medical imaging; it can be used for basic image analysis and measurement tasks.

#### NMQC Toolkit

In NMQC Toolkit image uniformity analysis, the tool performed multiple tasks to ensure adherence to the NEMA requirements. First, it inspected the pixel size to confirm its alignment within the NEMA-specified range of 6.4 mm ± 30% (ranging from 4.48 to 8.32 mm). Second, if the pixel size exceeded this range, an automatic matrix size rescaling procedure (using the sum method) would be initiated. In instances where the pixel size was smaller than NEMA's recommended minimum value, the adjacent detector pixels were combined to yield an effective pixel size within the specified range. However, it is crucial to note that acquiring uniformity images with a pixel size significantly exceeding the maximum value recommended by NEMA is discouraged. In such cases, a warning would be generated, and pixel size reduction would not be executed.

#### Fiji (ImageJ)

Fiji was not specifically developed for dedicated QC and testing purposes in nuclear medicine modalities. Nevertheless, it has gained widespread acceptance in the field of image analysis and various applications. It is important to note that, in this study, Fiji analysis adhered to the guidelines outlined in the NEMA standards. Fiji was solely employed for extracting pixel values from the uniformity image. Before pixel value extraction, the uniformity image underwent filtering using the convolution kernel, described in Equation 1 . 27 Following this, the uniformity analysis was carried out through calculations performed in Microsoft Excel.



During the calculation, only non-zero-pixel values were taken into account, excluding zero-pixel values at the periphery of the image. Using Microsoft Excel, integral, x-differential, and y-differential uniformities were computed based on the equations provided in NEMA Standards Publication NU-1 2018. Mathematically, the two equations seem similar ( Eq. 2 ). However, the integral uniformity was determined by calculating the difference between the maximum and the minimum pixel values within the respective fields of view (FOVs). In contrast, the differential uniformity involved finding the difference between the maximum and minimum pixel values within a set of five contiguous pixels in a row or column. 27



#### NM Toolkit

The NM Toolkit facilitates the automated assessment of uniformity analysis. To execute the analysis, the Digital Imaging and Communications in Medicine (DICOM) image was uploaded in the “Image Folder” and chosen for analysis. Subsequently, by simply clicking on the “NEMA Uniformity” option, the software displayed the postprocessed intrinsic uniformity image along with the test results.

### Statistical Analyses

This study used nonparametric Mann–Whitney *U* test to ascertain whether the differences between the integral and differential uniformities, obtained using integrated QC analysis software and three distinct types of free QC analysis software were statistically significant. To assess the agreement between the software, a Bland–Altman regression approach was employed as the computed data did not exhibit a normal distribution. 28 29

## Results

**Fig. 2:**
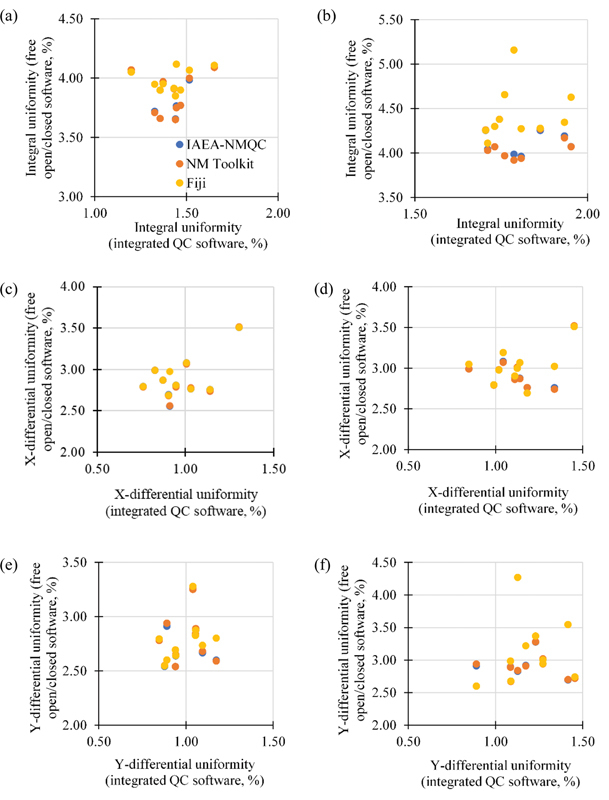
Comparison of the uniformities computed using integrated QC software and free QC software. ( **a, b** ) Integral uniformity. ( **c, d** ) X-differential uniformity. ( **e, f** ) Y-differential uniformity. Left panels: central field of view. Right panels: uniform field of view.

The intrinsic uniformity computed by integrated QC software of Discovery NM/CT 670 Pro scanner and the other three free open/closed software packages (NMQC Toolkit, Fiji, and NM Toolkit) for both central field of view (CFOV) and UFOV are illustrated in Fig. 2 . Percentage deviation was estimated in terms of percentage difference with respect to the uniformity values computed by the SPECT integrated QC software. Minor deviations between the integrated QC software and free open/closed software were observed, less than 3% for the integral uniformities and less than 2% for differential uniformities (see Table 2 ).

**Table 2:** Comparison of the uniformity, presented as a percentage deviation from the SPECT integrated QC software (standard deviation)

		NMQC Toolkit	Fiji	NM Toolkit
Integral uniformity (%)	CFOV	2.46 (0.18)	2.56 (0.13)	2.44 (0.19)
	UFOV	2.34 (0.19)	2.64 (0.30)	2.31 (0.18)
x-differential (%)	CFOV	1.91 (0.21)	1.96 (0.19)	1.91 (0.21)
	UFOV	1.84 (0.22)	1.90 (0.21)	1.83 (0.23)
y-differential (%)	CFOV	1.77 (0.24)	1.79 (0.18)	1.78 (0.24)
	UFOV	1.69 (0.26)	1.93 (0.50)	1.69 (0.27)

Abbreviations: CFOV, central field of view; QC, quality control; SPECT, single photon emission computed tomography; UFOV, useful field of view.

### Agreement between the Estimated Uniformity

The significant difference between the intrinsic uniformity computed by integrated QC software and each free software was confirmed by the Mann–Whitney *U* test ( *z* = −3.782 to −3.780, *p* < 0.001; Table 3 ). The other results were purposely not presented here due to the minor difference with the data presented here. In this study, the free software consistently resulted in higher uniformity compared to integrated QC software, presented by the higher mean rank (mean rank free software = 15.5, mean rank integrated QC software = 5.5).

**Table 3:** Mann–Whitney *U* test for uniformity analysis (integral and differential uniformity)

Variables	Group ( *N* )	Mean rank	*Z*	Sig.
Uniformity (%)	Integrated QC software (10)	5.5	−3.782 to −3.780	<0.001
	Free open/closed software (10)	15.5		

Note: The same outcomes were obtained for all free open/closed software compared to integrated QC software.

**Fig. 3:**
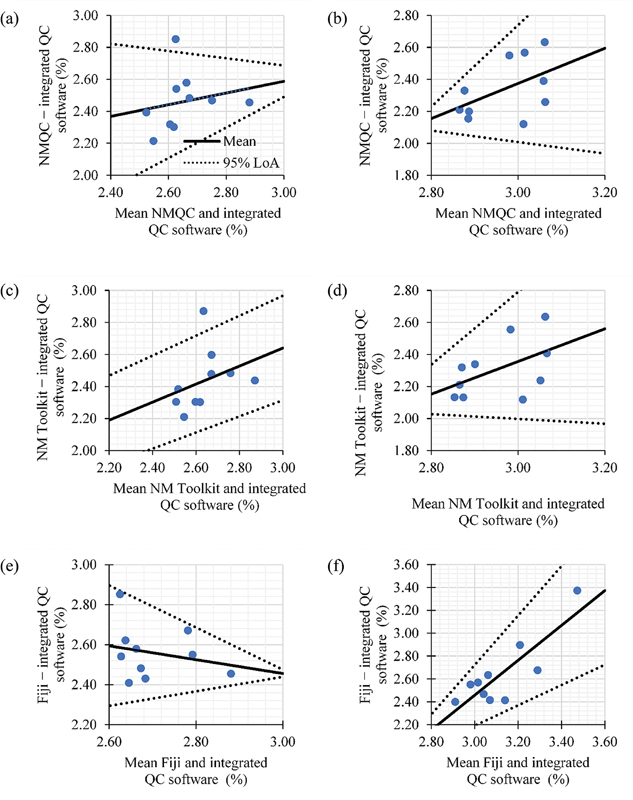
Bland–Altman regression-based limits of agreement for integral uniformity computed between ( **a, b** ) integrated quality control (QC) software and NMQC Toolkit; ( **c, d** ) integrated QC software and NM Toolkit; ( **e–f** ) integrated QC software and Fiji. A good level of agreement was observed, with the data falling within the 95% limits of agreement. The *solid line* indicates the mean difference (bias), and the *dotted lines* represent 95% limit of agreement. Left panels: central field of view. Right panels: uniform field of view.

Figs. 3^4^,5 present the regression-based limits of agreement for intrinsic uniformity in both CFOV and UFOV between the integrated QC software and free open/closed software. A good level of agreement was observed between the data. Most of the measurements were observed to be within the 95% limits of agreement, which were plotted as the upper and lower limits ( *dotted line* ). Observation of the slopes of the regression lines demonstrated that they are not exactly 45 degrees, indicating one method measured proportionately more or less than the other method. In this study, the free open/closed source QC analysis software resulted in higher percentages of intrinsic uniformity than the values computed by the integrated QC software in Discovery NM/CT 670 Pro.

**Fig. 4:**
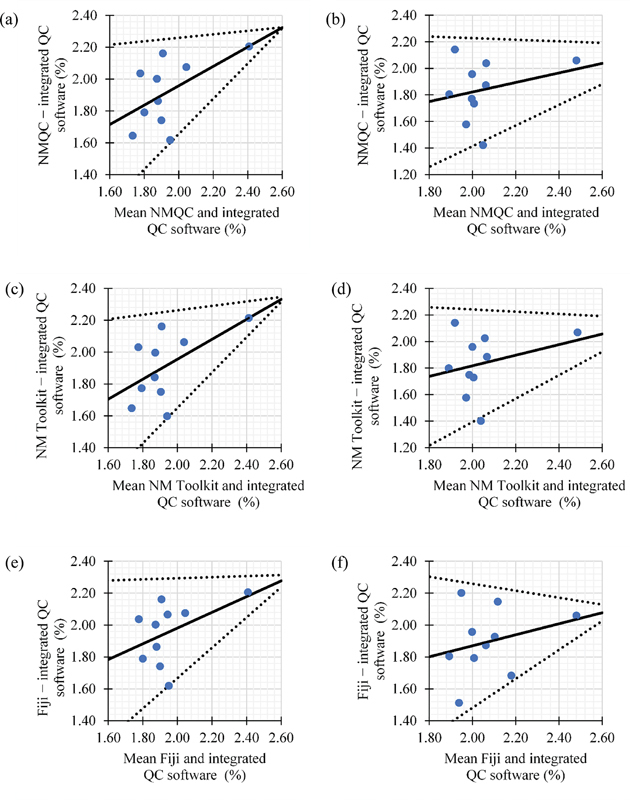
Bland–Altman regression-based limits of agreement for x-differential uniformity computed between ( **a, b** ) integrated quality control (QC) software and IAEA-NMQC Toolkit; ( **b, c** ) integrated QC software and NM Toolkit; ( **e, f** ) integrated QC software and Fiji. A good level of agreement was observed, with the data falling within the 95% limits of agreement. The *solid line* indicates the mean difference (bias), and the *dotted lines* represent 95% limit of agreement. Left: central field of view. Right panels: uniform field of view.

**Fig. 5:**
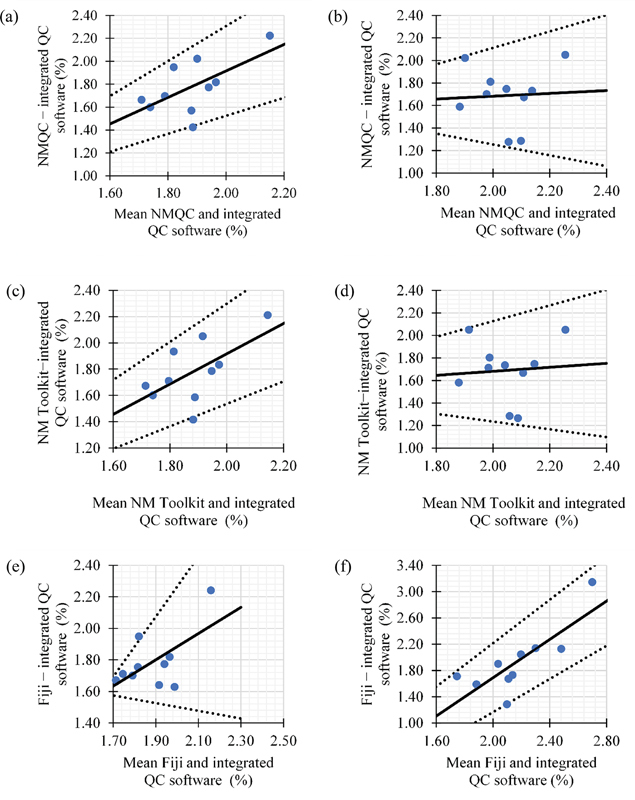
Bland–Altman regression-based limits of agreement for y-differential uniformity computed between ( **a, b** ) integrated quality control (QC) software and IAEA-NMQC Toolkit; ( **b, c** ) integrated QC software and NM Toolkit; ( **e, f** ) integrated QC software and Fiji. Left panels: A good level of agreement was observed, with the data falling within the 95% limits of agreement. The *solid line* indicates the mean difference (bias), and the *dotted lines* represent 95% limit of agreement. Left panels: central field of view. Right: uniform field of view.

## Discussion

SPECT, in comparison to planar imaging, offers superior localization of the radioactivity distribution. However, it faces challenges related to nonuniformity stemming from the back projection of data during image reconstruction. 30 Therefore, to ensure the optimal functioning of a SPECT system, various QC procedures, coupled with correction techniques, are implemented to address and enhance its performance. One of the critical factors to consider is the uniformity of the system itself, measuring its ability to generate a uniform image when exposed to uniform gamma rays. While some accept smaller nonuniformity within the 1 to 3% range, 31 our SPECT/CT allows for a slightly larger value, less than 5%. However, even a minor flaw in detector uniformity, as minimal as a 3% disparity, can lead to noticeable artifacts in the reconstructed image. 3 In line with this, some study suggests considering every component of the SPECT or gamma camera system for proper uniformity correction, including external factors such as the contribution from the collimator. 32

In this study, acquisition of uniformity image strictly adhered to the NEMA recommendations with the goal of assessing variations resulting solely from different analysis tools. The standard protocol for the uniformity assessment typically follows the NEMA guidelines, which provide standard recommendations. According to the NEMA guidelines, the assessment should use the energy window recommended by the manufacturer for the selected acquisition protocol, as the energy window significantly influences flood image uniformity. In ^99m^ Tc imaging, 20% photopeak window is commonly used, as studies have shown that a range of 15 to 20% yields optimal uniformity. Deviating from this range, either with a lower or higher photopeak window, has been demonstrated to associate with marked changes in uniformity. 2 In addition to that, to synchronize readings from two detectors, strict adherence to NEMA recommendations is crucial, ensuring a minimum of 10,000 counts collected in the center pixel of the image.

Uniformity assessment in this study resulted in nonuniformity of less than 5%, irrespective of the analysis tools used, thus complying with recommendations. 30 However, the difference between the data and the factors associated with it should be understood. Discrepancies were noted with respect to the QC software integrated into the scanner system, with greater nonuniformity estimated by the free open/closed software ( Table 2 and Fig. 2 ), aligning with previous findings. 13 This significant difference could be attributed by two factors. First, it is attributed to the correction methods applied by the SPECT system on the acquired image. Some gamma camera manufacturers, as per the IAEA, use uniformity correction, in addition to energy and linearity corrections, to correct for residual nonuniformity and collimator flaws. 8 In this study, the intrinsic uniformity computed by the integrated QC software was based on images that underwent postprocessing with uniformity correction by its system. Previously, a study highlighted the difference in intensity profiles of a flood-field image before and after uniformity correction, emphasizing the improvement in the standard deviation of the profile after correction. 6 However, extracting these postprocessed images from our system was not possible. For the analysis using independent software, a raw intrinsic uniformity image was utilized, and these images were normalized through convolution with the NEMA-defined kernel. 27 Hence, the difference between the integrated QC software and other free software was expected. Second, it could be attributed to the pixel size used during the analysis. 13 33 Some analysis tools like NM toolkit used default pixel size, for which alteration is not possible. Even though the NEMA allows pixel size in the range of 4.48 to 8.32 mm, the wide range of this value is believed to affect the pixels counts due to the smoothing effects, subsequently influencing the uniformity estimation. 13

A comparison of the free open/closed software shows that Fiji resulted in larger deviation from the integrated QC software (albeit <3% deviation and the majority of the computed values are overlapping each other), probably due to manual analysis done in Fiji. Fiji involved importing pixel values to Excel for computation (with reference to the NEMA guidelines), while others were automated computations, which display the finalized results without much, if any, elaboration on them. Therefore, Fiji analysis could be subjected to random and systematic errors due to the inherent variability in any sampling process during pixel selection process. Although the estimated nonuniformity was less than 5% and compliant with recommendations, 30 understanding the differences between the data and associated factors is crucial.

The Bland–Altman regression-based limit found in this study indicates good agreement between the integrated QC software and free open/closed source software in intrinsic uniformity analysis. Most measurements fell within the 95% limit of agreement, although there was at most one outlier for some plots ( Fig. 3a and c ). The outliers observed were in integral uniformity, as its assessment was performed for the entire flood to assess the overall performance of the system. However, integral uniformity lacks detailed spatial information and may not be sensitive to localized or small-scale variations. Pixel averaging performed on the image can mask variations at the pixel level and might not be sensitive to small changes in intensity. When observing the slopes of the regression lines, they are not exactly 45 degrees, indicating one method measured proportionately more or less than the other method. 34 In this study, the free open/closed QC analysis software resulted in higher percentages of intrinsic uniformity than the integrated QC analysis software developed by the vendor of Discovery NM/CT 670 Pro QC analysis software.

## Conclusion

Manufacturers have responded to the challenges in routine QC testing by equipping recent gamma camera systems with built-in QC analysis software, which has proven to be highly efficient. This study aimed to assess the agreement between uniformity analysis computed by the integrated QC software developed by manufacturers and the analysis performed by independent QC software. The data presented in this study are, however, limited to a single SPECT/CT scanner, the GE Discovery NM/CT 670 Pro system. Based on regression-based limits of agreement analyses, it is concluded that the intrinsic uniformity computed by the integrated QC software exhibits a good level of agreement with the data computed by the NMQC Toolkit, NM Toolkit, and Fiji. It is also affirmed that the QC analysis processes of the integrated QC software align well with the standard QC analysis protocols recommended by the NEMA. In conclusion, the uniformity analysis performed by the integrated QC software is reliable and yields values closely aligned with the NEMA standards. The comparison serves as a benchmark for the performance of the integrated QC software in assessing uniformity. The findings aid practitioners and researchers in making informed decisions about the selection of analysis tools for uniformity assessments. Further analysis incorporating different models of SPECT/CT scanners would be beneficial to validate the findings across a broader range of systems and enhance the generalizability of the results. By contributing to the ongoing efforts in standardization within the field, this work helps establish best practices for uniformity assessment, harmonizes procedures across different imaging centers, promotes consistency, and facilitates multicenter studies and comparisons.

## References

[1] Zhang R Wang M Zhou Y Impacts of acquisition and reconstruction parameters on the absolute technetium quantification of the cadmium-zinc-telluride-based SPECT/CT system: a phantom study EJNMMI Phys 2021 8 01 66 34568990 10.1186/s40658-021-00412-4 PMC8473509

[2] Abdelhalim M AK Rizk R AM Farag H I Reda S M Effect of energy window width on planer and SPECT image uniformity J King Saud Univ Sci 2009 21 02 145 150

[3] Seret A Bleeser F Intrinsic uniformity requirements for pinhole SPECT J Nucl Med Technol 2006 34 01 43 47 16517968

[4] Mezzenga E Sarnelli A Bellomo G DiFilippo F P Palestro C J Nichols K J Quantification of SPECT concentric ring artifacts by radiomics and radial features Appl Sci 2022 12 05 2726

[5] Leong L K Kruger R L O'Connor M K A comparison of the uniformity requirements for SPECT image reconstruction using FBP and OSEM techniques J Nucl Med Technol 2001 29 02 79 83 11376099

[6] Cherry S R Sorenson J A Phelps M E Physics in Nuclear Medicine Philadelphia, PA Elsevier 2012

[7] Saha G B Physics and Radiobiology of Nuclear Medicine 4th ed. New York, NY Springer 2013

[8] International Atomic Energy Agency IAEA Quality Control Atlas for Scintillation Camera Systems Vienna, Austria International Atomic Energy Agency 2003

[9] Lopez B P Jordan D W Kemp B J Kinahan P E Schmidtlein C R Mawlawi O R PET/CT acceptance testing and quality assurance: executive summary of AAPM Task Group 126 Report Med Phys 2021 48 02 e31 e35 33320364 10.1002/mp.14656

[10] Pandey A K Sharma P D Kumar J P Calculating gamma camera uniformity parameters: beyond the vendor-specific protocol Indian J Nucl Med 2017 32 04 279 282 29142343 10.4103/ijnm.IJNM_67_17 PMC5672747

[11] Schunn C D Trafton J G The psychology of uncertainty in scientific data analysis New York, NY Springer 2013 461 483

[12] Cohen L Manion L Morrison K Research Methods in Education 8th ed. London Routledge 2018

[13] Edam A N Fornasier M R De Denaro M Sulieman A Alkhorayef M Bradley D A Quality control in dual head gamma-cameras: comparison between methods and software used for image analysis Appl Radiat Isot 2018 141 288 291 30122471 10.1016/j.apradiso.2018.07.027

[14] Ziada G Yousef Z Abdel-Azzem A A comparison between two methods of performing the daily intrinsic uniformity quality control test for gamma camera Egypt J Nucl Med 2010 2 01 40 54

[15] Vickery A Jørgensen T de Nijs R NEMA NU-1 2007 based and independent quality control software for gamma cameras and SPECT J Phys: Conf Ser 2011 317 12023

[16] Islam K Siraj M S Zaneb H Sohail A Qureshy A Assessment of intrinsic and extrinsic uniformity for dual head SPECT gamma camera J Med Phys Appl Sci 2022 7(5:23):1 8

[17] Rova A Celler A Hamarneh G Development of NEMA-based software for gamma camera quality control J Digit Imaging 2008 21 02 243 255 17393254 10.1007/s10278-007-9030-y PMC3043858

[18] Krizsan A K Kukuts K Al-Muhanna W Performance evaluation of a novel multi-pinhole collimator on triple-NaI-detector SPECT/CT for dedicated myocardial imaging EJNMMI Phys 2023 10 01 24 36964406 10.1186/s40658-023-00541-y PMC10039219

[19] Valentim F O Coelho B P Miot H A Follicular thyroid lesions: is there a discriminatory potential in the computerized nuclear analysis? Endocr Connect 2018 7 08 907 913 29973373 10.1530/EC-18-0237 PMC6063880

[20] Hirtl A Bergmann H Knäusl B Beyer T Figl M Hummel J Technical Note: Fully-automated analysis of Jaszczak phantom measurements as part of routine SPECT quality control Med Phys 2017 44 05 1638 1645 28186647 10.1002/mp.12150

[21] Sousa C LD Carolino E Figueiredo S Imagej's contribution to left ventricular segmentation in myocardial perfusion imaging Nucl Med Biomed Imaging 2017 2 01 1 7

[22] Oktaviana A T Pawiro S A Siswatining T Soejoko D S Preliminary study of ring artifact detection in SPECT imaging using Jaszczak phantom J Phys Conf Ser 2019 1248 12030

[23] Bordonne M Chawki M B Marie P Y High-quality brain perfusion SPECT images may be achieved with a high-speed recording using 360° CZT camera EJNMMI Phys 2020 7 01 65 33146804 10.1186/s40658-020-00334-7 PMC7642149

[24] Caobelli F Kaiser S R Thackeray J T IQ SPECT allows a significant reduction in administered dose and acquisition time for myocardial perfusion imaging: evidence from a phantom study J Nucl Med 2014 55 12 2064 2070 25413138 10.2967/jnumed.114.143560

[25] Rasband WImage J. National Institutes of Health. Accessed 26 March 2023 at:http://imagej.nih.gov/ij/

[26] QA Benchmark, LLC. Accessed 26 March 2023 at:http://QABenchmark.com

[27] NEMA NEMA Standards Publication NU 1–2018: Performance Measurements of Gamma Cameras Rosslyn, VA National Electrical Manufacturers Association 2019

[28] Bland J M Altman D G Measuring agreement in method comparison studies Stat Methods Med Res 1999 8 02 135 160 10501650 10.1177/096228029900800204

[29] Giavarina D Understanding Bland Altman analysis Biochem Med (Zagreb) 2015 25 02 141 151 26110027 10.11613/BM.2015.015 PMC4470095

[30] Zanzonico P Routine quality control of clinical nuclear medicine instrumentation: a brief review J Nucl Med 2008 49 07 1114 1131 18587088 10.2967/jnumed.107.050203 PMC2703015

[31] Prekeges J Nuclear Medicine Instrumentations Sudbury, MA Jones and Bartlett Learning 2011

[32] Bolstad R Brown J Grantham V Extrinsic versus intrinsic uniformity correction for γ-cameras J Nucl Med Technol 2011 39 03 208 212 21795372 10.2967/jnmt.110.084814

[33] Islam K Murad S Siraj M S Qureshy A Acceptance testing and routine testing of dual head SPECT gamma camera at a new cancer hospital J Med Phys Appl Sci 2022 7 05 22

[34] Hoffman J I Biostatistics for Medical and Biomedical Practitioners 2nd ed. London Academic Press 2019

